# Quantitative Structure-Activity Relationship Studies on Indenoisoquinoline Topoisomerase I Inhibitors as Anticancer Agents in Human Renal Cell Carcinoma Cell Line SN12C

**DOI:** 10.3390/ijms13056009

**Published:** 2012-05-18

**Authors:** Yi Zhi, Jin Yang, Shengchao Tian, Fang Yuan, Yang Liu, Yi Zhang, Pinghua Sun, Bo Song, Zhiwen Chen

**Affiliations:** 1 Urology Center, Southwest Hospital, Third Military Medical University, Chongqing 400038, China; E-Mails: mtzhi@yahoo.com.cn (Y.Z.); shengchaotian@163.com (S.T.); iotayy@126.com (F.Y.); liuyangjfj@yeah.net (Y.L.); 2 Department of Cell Biology, Third Military Medical University, Chongqing 400038, China; E-Mails: yangjing_cq@yahoo.cn (J.Y.); zhy3210@163.com (Y.Z.); 3 Guangdong Province Key Laboratory of Pharmacodynamic Constituents of Traditional Chinese Medicine and New Drugs Research, College of Pharmacy, Jinan University, Guangzhou 510632, China; E-Mail: biochemdoctor@sina.com

**Keywords:** CoMFA, CoMSIA, QSAR, indenoisoquinoline, Top1 inhibitors

## Abstract

Topoisomerase I is important for DNA replication and cell division, making it an attractive drug target for anticancer therapy. A series of indenoisoquinolines displaying potent Top1 inhibitory activity in human renal cell carcinoma cell line SN12C were selected to establish 3D-QSAR models using CoMFA and CoMSIA methods. Internal and external cross-validation techniques were investigated, as well as some measures taken, including region focusing, bootstrapping and the “leave-group-out” cross-validation method. The satisfactory CoMFA model predicted a *q*^2^ value of 0.659 and an *r*^2^ value of 0.949, indicating that electrostatic and steric properties play a significant role in potency. The best CoMSIA model, based on a combination of steric, electrostatic and H-bond acceptor descriptors, predicted a *q*^2^ value of 0.523 and an *r*^2^ value of 0.902. The models were graphically interpreted by contour plots which provided insight into the structural requirements for increasing the activity of a compound, providing a solid basis for future rational design of more active anticancer agents.

## 1. Introduction

Kidney cancer is among the 10 most frequently occurring cancers in western communities. Globally, about 270,000 cases of kidney cancer are diagnosed yearly and 116,000 people die from the disease. Renal cell carcinoma (RCC) accounts for approximately 90% of all kidney cancers and its incidence is on the rise [1,2]. Localized RCC is curable with surgery but a third of patients are diagnosed with metastatic RCC, which is difficult to treat and is generally resistant to conventional radiotherapy, chemotherapy and endocrine therapy. The median survival for patients with metastatic RCC is 10–12 months [3]. Despite a minority of patients with metastatic disease benefiting from cytokine immunotherapy, a need still exists for developing more effective novel anti-renal cell carcinoma agents.

Human topoisomerase type I (Top1) is a member of the topoisomerase family of enzymes that resolve the topological problems associated with DNA supercoiling during various essential cellular processes [4–6]. It forms a covalent link with the 3′-end of the cut DNA strand in the Top1-DNA cleavage complex at its catalytic tyrosine 723 residue, relieving torsional strain in DNA via reversible single-strand nicks [7,8]. Top1 is important for the successful replication, transcription and recombination of DNA, as well as chromatin remodeling, making it an attractive drug target for anticancer therapy. Camptothecin, isolated and identified in 1966, was the first Top1 inhibitor [9]. Camptothecin derivatives irinotecan and topotecan approved by the Food and Drug Administration (FDA) validate Top1 as a therapeutic target for anticancer drug development [10]. In practice, these Top1 inhibitors exert a promising anticancer effect in the treatment of renal cell carcinoma. For instance, clinically relevant concentrations of topotecan-induced apoptosis in RCC cell lines work more effectively than 5-FU [11]. In addition, combination therapy using topotecan and survivin-specific siRNA could show a synergistic effect and offer an attractive approach for the treatment of advanced renal cancer [12,13]. In clinical practice, the use of a novel combination of irinotecan, cisplatin and mitomycin (IPM chemotherapy) produce symptomatic relief for a majority of patients with renal cancer following failure of cytokine immunotherapy [14]. However, these camptothecin derivatives are not ideal drug molecules, suffering from pharmacokinetic problems, inherent instability due to lactone ring opening and rapid reversibility of the cleavage complexes after drug removal [15,16]. There is a present need for the development of noncamptothecin Top1 inhibitors as anticancer agents. Recently, a number of analogs of the indenoisoquinolines have been reported as novel anticancer agents [17–19]. The indenoisoquinoline Top1 inhibitors were examined for antiproliferative activity against different cancer cell lines. The results indicate that these novel noncamptothecin Top1 inhibitors could be potential agents for the treatment of a variety of cancers, including renal cancer. Among these derivatives, two indenoisoquinolines have been selected currently for clinical development by the NCI: NSC 725776 and NSC 724998 [20]. Both exert antiproliferative activity in submicromolar concentrations in cultured human cancer cell lines.

The three-dimensional quantitative structure-activity relationship (3D-QSAR) techniques, including comparative molecular field analysis (CoMFA) [21] and comparative similarity indices analysis (CoMSIA) [22] are useful methods of ligand-based drug design used to correlate physicochemical descriptors from a set of related compounds to their known molecular activity or molecular property values [23]. These computational techniques incorporate 3D information for the ligands and have been proved particularly helpful in the design of novel and more potent inhibitors. The application of QSAR methodology to the indenoisoquinoline derivatives hasn’t been reported. The satisfactory QSAR models on 48 indenoisoquinoline topoisomerase I inhibitors for their anti-renal cell carcinoma activities [18,19] provide a solid basis for future rational design of more active agents.

## 2. Results and Discussion

### 2.1. CoMFA Analysis

**Figure 1:**
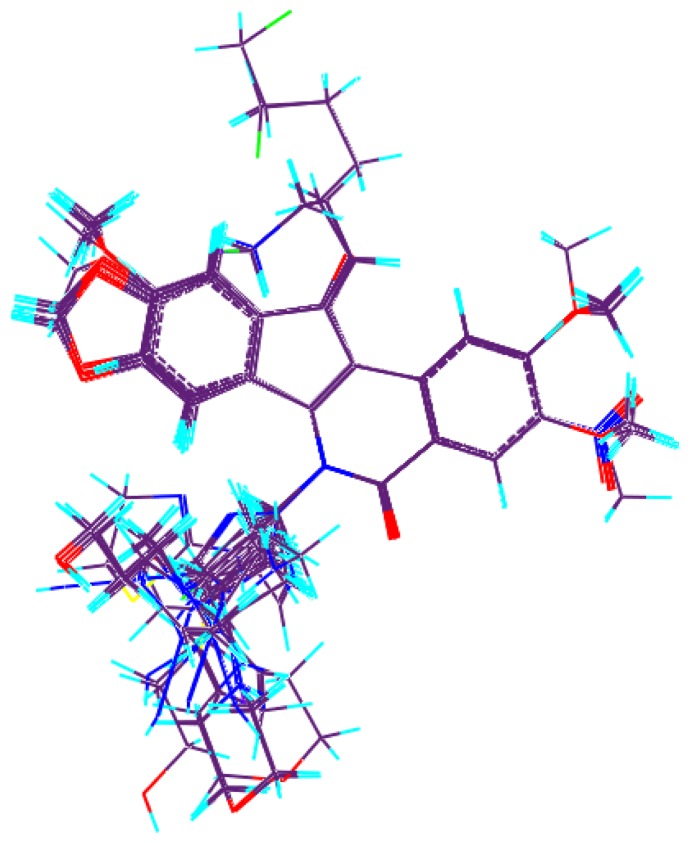
Molecular alignment of indenoisoquinoline derivatives.

**Figure 2:**
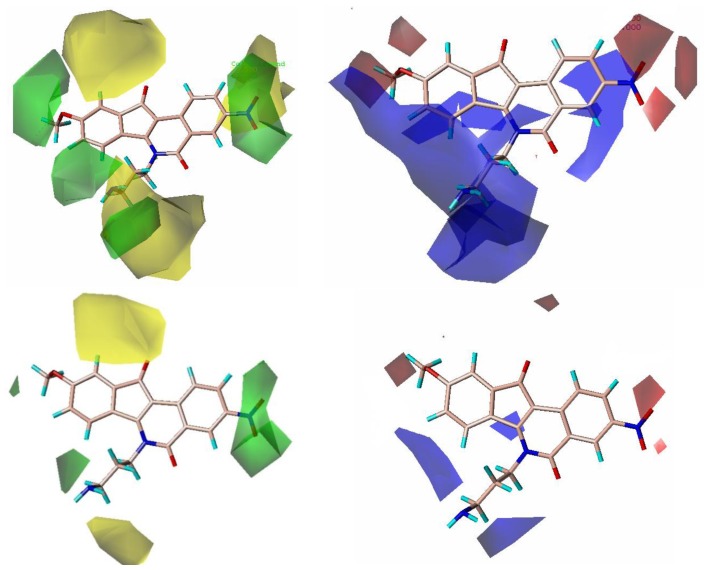
Region focusing. The CoMFA field calculations are shown for compound **20** before (Upper) and after (Lower) region focusing. Steric fields (Left): Green fields indicate steric bulk favored, yellow fields indicate steric bulk disfavored. Electrostatic fields (Right): Blue fields indicate electropositive groups favored, red fields indicate electronegative groups favored.

**Figure 3:**
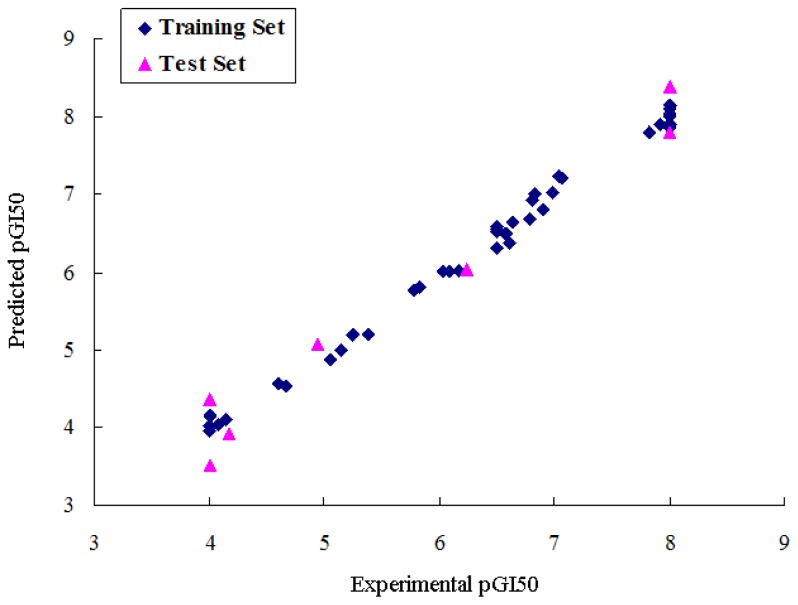
Graph of experimental *versus* predicted pGI50 of the training set and the test set using the CoMFA model.

The compound **20**, one of the most active molecules, was selected as the template and the isoquinoline ring as the common structure for alignment (Figure 1). The CoMFA model provided a cross-validation *q*^2^ value of 0.602 with 5 components, an *r*^2^ value of 0.925 and an *F*-test value of 66.709 (Table 1). Region focusing resulted in the better CoMFA model which showed a significant increase from 0.602 to 0.659 for the internal validity, 0.632 to 0.680 for group cross-validation, 0.790 to 0.826 for test set activity predictions, and from 0.925 to 0.949 for the non-validated *r*^2^ (Table 1). Figure 2 shows CoMFA fields for molecule **20** before and after region focusing. The activity values predicted for the test set are in good agreement with the experimental values (Figure 3) and the *r*_pred_^2^ value of 0.826 further confirms the reliability and accuracy of the model. The electrostatic and steric field contributions to the final model were 58.7% and 41.3%, respectively.

### 2.2. CoMSIA Analysis

**Figure 4:**
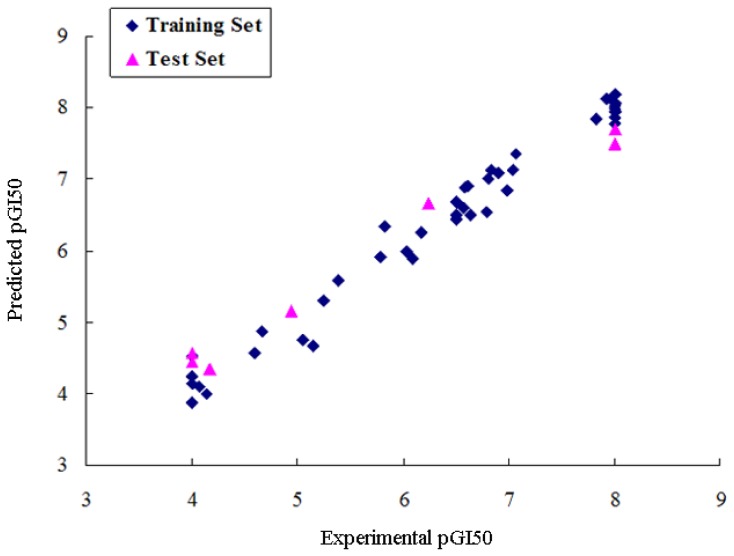
Graph of experimental *versus* predicted pGI50 of the training set and the test set using the best CoMSIA model 5.

Twelve CoMSIA models were generated using combinations of 2, 3, 4, and all 5 descriptors as shown in Table 2. Model 5, based on steric, electrostatic and H-bond acceptor fields, was found to be the most accurate, yielding a *q*^2^ value of 0.523 and an *r*^2^ value of 0.902. The Group cross *q*^2^ value of 0.524, bootstrapped value of 0.906 ± 0.023 and test set *r*^2^ value of 0.704 further approve the best CoMSIA model 5. The predicted values are closely consistent with the experimental data (Figure 4). The steric field explains 13.4% of the variance, the electrostatic field for 47.9% and the H-bond acceptor field for 38.7% of the variance.

### 2.3. CoMFA Contour Maps

**Figure 5:**
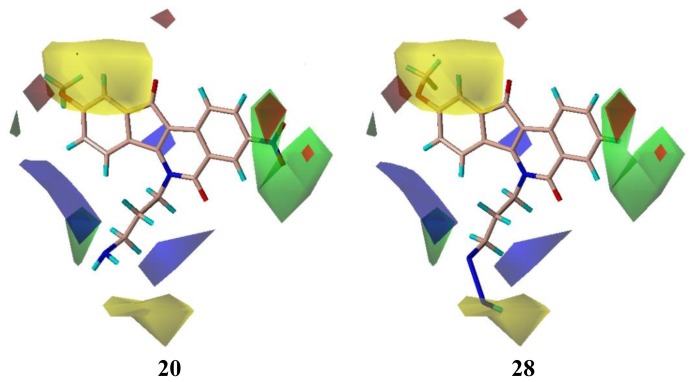
CoMFA contour maps of the highest active compound **20** and the lowest active compound **28**.

The results of 3D-QSAR models are presented in the contour coefficient maps as shown in Figure 5. Its steric interaction is denoted by green and yellow contours. Both a large green contour and a large yellow contour were located near the end of the side chain linking to the nitrogen atom of the isoquinoline ring of target compounds, indicating that steric fields did not play an important role in this region. This may be the reason why compounds **20** and **28** with almost the same chains showed the most and lowest activities, respectively. Similarly, compounds **1**, **24**, **28**, **31** and **32** showed lower activity while compounds **3**, **6**, **17**, **19** and **29** are more potent. Two large green and two small red contours around the 3-position of the isoquinoline ring suggest that bulky and electron-withdrawing substituents are required in this region to increase activity. This is possibly the reason why compound **39** with the substitution of nitro group showed 24.5 times more potency than its corresponding mother compound **40**, likewise **41** is 67.6 times greater than **42**. A small red contour located near carbonyl group at position-11 of compound **20** indicates that electron-withdrawing groups are preferred in this region. This is why the compounds **43**–**47**, whose carbonyl group at position-11 was replaced by other electron-donating groups, are less potent. A small red contour near the methoxyl substituted at position-9 of compound **20** can be interpreted that groups with an electron-withdrawing factor are desired to increase the activity, and that is why compound **20** with the methoxyl group at position-9 is almost 7000 times more potent than its mother compound **24**, also compounds **36** and **9** are far more potent than corresponding **35** and **33**, respectively. A large yellow contour around position-1 signifies that the hydrogen atom must not be substituted.

### 2.4. CoMSIA Contour Maps

**Figure 6:**
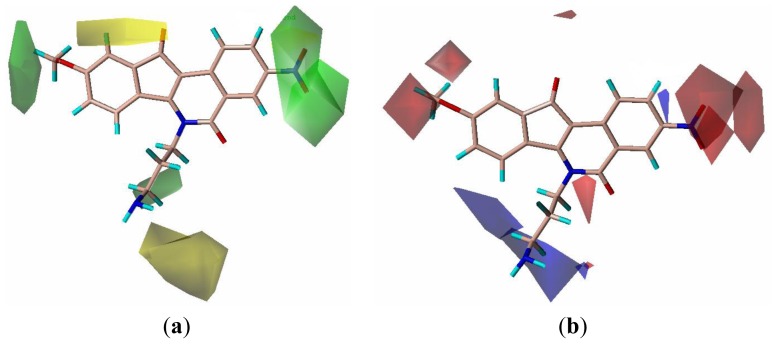

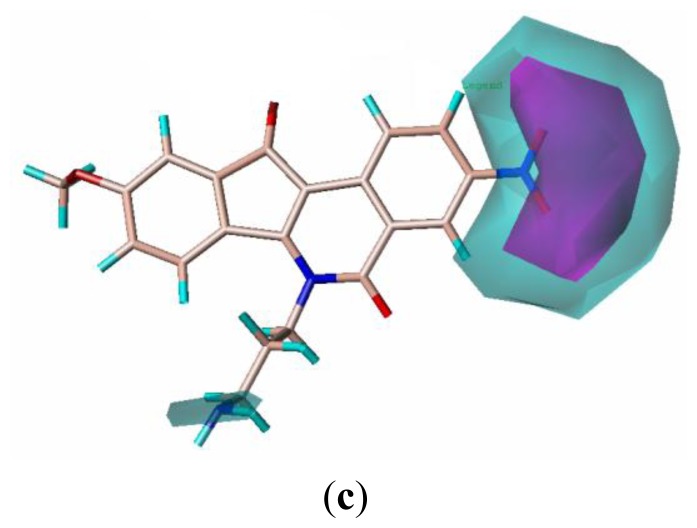
CoMSIA fields. The CoMSIA fields from model 5 are shown with active compound **20**; (**a**) Steric fields: green indicates steric bulk favored, yellow indicates bulk disfavored; (**b**) Electrostatic fields: blue indicates electropositive groups favored, red fields indicate electronegative groups favored; (**c**) H-bond acceptor fields: magenta indicates acceptor favored, cyan disfavored.

The best CoMSIA model contour maps of the most active analog are shown in Figure 6. Its steric and electrostatic contour plots (Figure 6a,b) correlate well with the CoMFA contour maps described above. Hydrogen-bond acceptor contour maps are shown in Figure 6c. Hydrogen bond acceptor-favored regions are represented by magenta contours and unfavorable regions by cyan contours. One large magenta polyhedron is visible around the 3-position of the isoquinoline ring of compound **20**, indicating that hydrogen-bond acceptor groups such as nitro, methoxyl group are very important for compound activity. Large cyan polyhedra around 2,4-positions of the isoquinoline ring and around the end of the side chain adjacent to the nitrogen atom of the isoquinoline ring can be interpreted as disfavoring hydrogen-bond acceptor groups in these regions.

### 2.5. Design of New Inhibitors

Based on the structure–activity relationship obtained by present 3D-QSAR models, a series of new inhibitors was designed and predicted (Table 3). With the most active molecule **20** in the training set used as the parent compound, some hydrogen-bond donors such as amino, hydroxyl and thiol were introduced at 3′ or 4′-position of the heterocycle appended to the lactam side chain, and some bulky and electron-withdrawing groups, such as nitro and cyan, introduced at the 3-position. Most (pGI50 > 8.5) greatly enhanced inhibitory activity in comparison to **20** (pGI50 = 8.145). In particular, compound **20-7** showed the strongest activity with its predicted pGI50 (9.029). Other compounds also exhibited good predicted activity as well as compound **20**.

## 3. Experimental Section

### 3.1. Data Set

Forty-eight compounds investigated in the present study were taken from the published works of Morrell A. and co-workers [18,19]. The structures of the molecules and their biological data obtained by Morrell A. *et al*. are given in Tables 4,5. For convenience, we have converted the cytotoxicity GI50 values of topoisomerase inhibitors in renal carcinoma cell line SN12C to their negative logarithm (pGI50) values, which have a span of 4.0 log units from 4.00 to 8.00, providing a broad and homogeneous data set for 3D-QSAR study (see Table 5) [24,25]. Seven compounds were randomly selected as the test set, based on the structural and active diversities with the remaining 41 compounds as the training set.

### 3.2. Molecular Alignment

Compared to probe atom type, lattice shifting step size and overall orientation of the aligned compounds, a good alignment is the most important element for CoMFA and CoMSIA analysis [26], and the alignment rules will directly determine the quality and the predictive ability of the model. The alignment was often performed in accordance with some rules, such as substructure overlap, pharmacophore overlap and docking [27] as soon as the active conformation was obtained by energy minimization using Powell method and Tripos standard force field [28]. Here, the isoquinoline ring with structural rigidity was selected as the common substructure to overlap and to align all of the molecules and the most active compound **20** was used as the alignment template. Alignment of all compounds was shown in Figure 1. It can be seen that all the compounds studied have similar active conformations.

### 3.3. Partial Least Squares (PLS) Analysis

To linearly correlate the 3D-QSAR fields to biological activity values, PLS analysis [29] was performed. It was firstly carried out by the leave-one-out (LOO) and leave-group-out (10 groups) cross-validation methods to determine cross-validated *r*^2^ (*q*^2^) values and the optimal number of components on the basis of the lowest standard error of prediction (SEP) and avoiding over-fitting the models. A higher component was accepted and used only when the *q*^2^ differences between two components were larger than 5%. Non-cross-validation was then performed to establish the final 3D-QSAR model with the values of conventional correlation coefficient (*r*^2^), standard errors of estimate (SEE), and *F* ratio between the variances of calculated and observed activities given.

The *q*^2^ has been a good indicator of the accuracy of actual predictions. In general, *q*^2^ values can be separated into three categories: *q*^2^ > 0.6 means a fairly good model, *q*^2^ = 0.4–0.6 means a questionable model, and *q*^2^ < 0.4 a poor model [30]. *q*^2^ is calculated as follows:

$$q^{2}=1-\frac{\sum {\left(Y_{obs}-Y_{pre}\right)}^{2}}{\sum {\left(Y_{obs}-Y_{mean}\right)}^{2}}$$

where, *Y*_obs_ = experimental activity, *Y*_pre_ = predicted activity, *Y*_mean_ = mean activity.

To further assess the robustness of the derived models, bootstrapping analysis (10 runs) was also utilized to calculate confidence intervals for the *r*^2^ and SEE [29,31]. The equation for SEE is given below.

$$SEE=\sqrt{\frac{PRESS}{n-c-1}}$$

Where *n* means number of compounds, *c* means number of components, and PRESS (predicted sum of squares) means ∑ (*Y*_obs_-*Y*_pre_)^2^.

### 3.4. Predictive Correlation Coefficient

*q*^2^ is a useful but not sufficient criterion for model validation, so an external test set (*r*_pred_^2^) [32] was claimed for the estimation of predictive ability. Equation of predictive values *r*_pred_^2^ is as follows:

$$r_{pred}^{2}=1-(PRESS/SD)$$

Therein, SD means the sum of squared differences between the measured activities of the test set and the average measured activity of the training set.

### 3.5. CoMFA Studies

Three-dimensional grid spacing was set at 2 Å in the *x*, *y*, and *z* directions and automatically generated to be a 3D cubic lattice that extended at least 4 Å beyond the van der Waals volume of all aligned molecules in all directions. Lennard-Jones potential and Coulomb potential were employed to calculate steric and electrostatic energies of each molecule using the Tripos force field [28], and the *sp*^3^-hybrized carbon atom with a +1 charge taken as the probe atom to determine the magnitude of the field values. The regression analysis was carried out using the partial least squares (PLS) method [29]. All energies that exceeded the cutoff value of 30 kcal/mol were replaced with 30 kcal/mol for the reduction of domination by large steric and electrostatic energies [33]. The column filtering was set to 2.0 kcal/mol and those lattice points whose energy variation was below this threshold were automatically omitted, consequently the signal-to-noise ratio was improved. The final model was developed with the optimum number of components to yield a non-cross-validated *r*^2^ value. Despite being unable to describe all of the binding forces, CoMFA is still a useful tool for QSAR analysis at the 3D level.

One method of 3D-QSAR optimization is known as region focusing [34], which may enhance or attenuate the contribution of the lattice points in a further analysis of a given CoMFA or CoMSIA region. Generally, region focusing can maximize the *q*^2^ value by rotating the extracted principal components, and give a new model with increased predictive power (*q*^2^), enhanced resolution, tighter grid spacing, and greater stability at a higher number of components.

### 3.6. CoMSIA Studies

CoMSIA is an extension of CoMFA on the same assumption that changes in binding affinities of ligands are related to changes in molecular properties represented by fields. Besides steric and electrostatic fields, three other different fields (hydrophobic, hydrogen bond donor, and hydrogen bond acceptor) are calculated in CoMSIA [35]. Moreover, a Gaussian function was introduced to determine the distance between the probe atom and the molecule atoms, and similarity indices inside and outside different molecular surfaces can be calculated at all grid points in CoMSIA. The equation used to calculate the similarity indices is as follows:

$$A_{F,K(j)}^{q}=\sum_{i}W_{probe,k}W_{ik}e^{-\alpha r_{iq}^{2}}$$

Where, *A* is the similarity index at grid point *q*, summed over all atoms *i* of the molecule *j* under investigation. *W*_probe, k_ is the probe atom with radius 1 Å, charge +1, hydrophobicity +1, hydrogen bond donating +1 and hydrogen bond accepting +1. *W*_ik_ is the actual value of the physicochemical property *k* of atom *i. r*_iq_ is the mutual distance between the probe atom at grid point *q* and atom *i* of the test molecule. *α* is the attenuation factor whose optimal value is normally between 0.2 and 0.4, with a default value of 0.3 [36,37].

## 4. Conclusions

In conclusion, our present studies have established predictive CoMFA and CoMSIA models that are quite reliable to efficiently guide further modification in the molecules for obtaining better drugs. Both of them provided good statistical results in terms of *q^2^* and *r*^2^ values, suggesting the significant correlations of molecular structures with its biological activities. Compared with CoMSIA, CoMFA provided a slightly better statistical model. The final CoMFA model has high internal validity (*q*^2^ above 0.5) and high predictive ability (test set *r*^2^ above 0.7). The 3D-QSAR results also revealed some important sites, where steric, electrostatic and hydrogen-bond acceptor modifications should significantly affect the bioactivities of these compounds. Thus, the results of the quantitative structure activity relationships (QSAR) studies give insight into how to design new inhibitors, and it can be expected that these novel derivatives could be more active anticancer agents in the treatment of renal cell carcinoma as well.

## Figures and Tables

**Table 1:** Statistical results of CoMFA and best CoMSIA models.

Statistical results	CoMFA(before region focusing)	CoMFA (after region focusing)	CoMSIA (Model 5)
*PLS* statistics *			
LOO cross *q*^2^/SEP #	0.602/0.855	0.659/0.781	0.523/0.923
Group cross *q*^2^/SEP	0.632/0.822	0.680/0.757	0.524/0.922
Non-validated *r*^2^/SEE ¤	0.925/0.367	0.949/0.334	0.902/0.436
*F*	66.709	84.997	64.275
*r*^2^_bootstrap_	0.918 ± 0.019	0.973 ± 0.020	0.906 ± 0.023
*S*_bootstrap_	0.387 ± 0.193	0.367 ± 0.135	0.373 ± 0.163
Optimal components	5	5	5
Field distribution%			
Steric	56.5	58.7	13.4
Electrostatic	43.5	41.3	47.9
H-bond acceptor			38.7
*r*^2^_pred_	0.790	0.826	0.704

* PLS = partial least squares,

# LOO= leave-one-out,

¤ SEE = standard errors of estimate.

**Table 2:** Results of CoMSIA models using combinations of the 5 field descriptors.

Model	Descriptors	LOO cross *q*^2^/SEP	Group cross *q*^2^/SEP	Bootstrap *r*^2^	Bootstrapped SEE	Non-validated *r*^2^/SEE
1	S and E	0.474/0.970	0.490/0.955	0.865 ± 0.043	0.479 ± 0.262	0.857/0.507
2	D and A	0.410/1.056	0.360/1.100	0.797 ± 0.066	0.599 ± 0.339	0.750/0.687
3	S, E and H	0.520/0.929	0.523/0.923	0.788 ± 0.044	0.593 ± 0.198	0.767/0.637
4	S, E and D	0.482/0.976	0.477/0.983	0.862 ± 0.034	0.496 ± 0.234	0.826/0.565
5	S, E and A	0.523/0.923	0.524/0.922	0.906 ± 0.023	0.373 ± 0.163	0.902/0.436
6	E, D and H	0.500/0.945	0.468/0.975	0.834 ± 0.055	0.528 ± 0.301	0.833/0.574
7	E, A and	H 0.511/0.923	0.500/0.933	0.757 ± 0.048	0.622 ± 0.296	0.765/0.639
8	S, E, D and A	0.519/0.927	0.535/0.938	0.922 ± 0.019	0.379 ± 0.169	0.827/0.556
9	S, E, D and H	0.503/0.942	0.560/0.886	0.834 ± 0.047	0.530 ± 0.274	0.816/0.574
10	S, E, A and H	0.521/0.925	0.533/0.892	0.785 ± 0.062	0.596 ± 0.321	0.808/0.585
11	S, D, A and H	0.453/0.996	0.484/0.987	0.870 ± 0.021	0.476 ± 0.174	0.833/0.562
12	S, E, D, A and H	0.502/0.956	0.519/0.940	0.879 ± 0.051	0.437 ± 0.251	0.899/0.445

**Table 3:** Results of CoMSIA models using combinations of the five field descriptors.

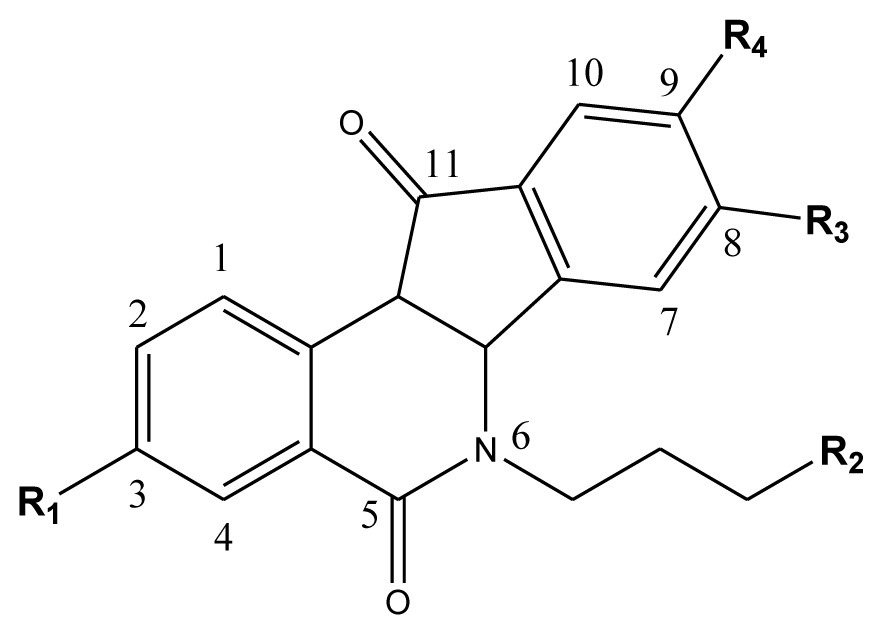						

No.	Substituents				Predicted pGI50	
	R_1_	R_2_	R_3_	R_4_	CoMFA	CoMSIA
20	NO_2_	NH_2_	H	OCH_3_	8.145	8.195
20-1	CN	NH_2_	H	OCH_3_	8.505	8.479
20-2	CN	NH_2_	OCH_3_	OCH_3_	8.134	8.065
20-3	CN	NH_2_	methylenedioxy		8.470	8.467
20-4	NO_2_	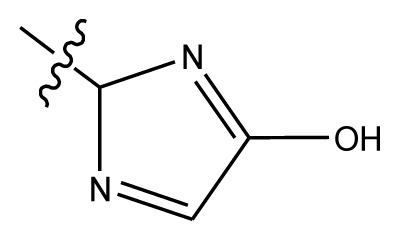	OCH_3_	OCH_3_	8.599	8.557
20-5	NO_2_	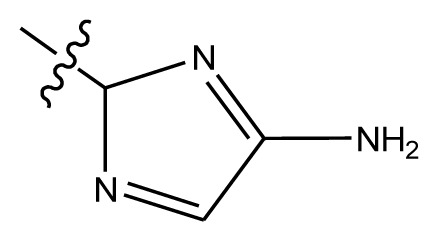	methylenedioxy		8.657	8.701
20-6	CN	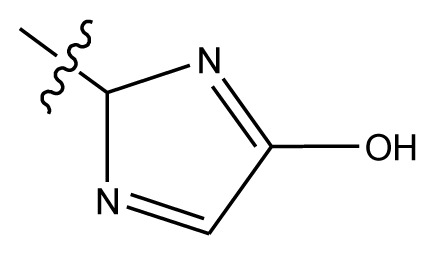	OCH_3_	OCH_3_	8.878	8.770
20-7	CN	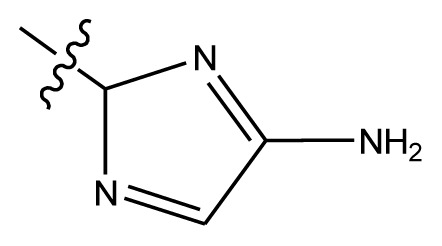	methylenedioxy		9.029	8.914
20-8	CN	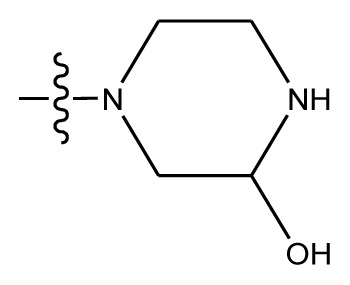	OCH_3_	OCH_3_	8.348	8.430
20-9	NO_2_	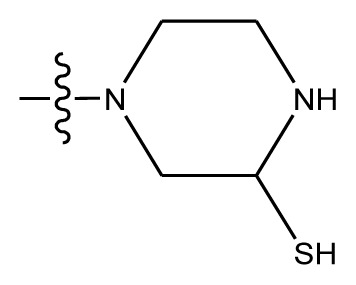	OCH_3_	OCH_3_	8.679	8.664
20-10	CN	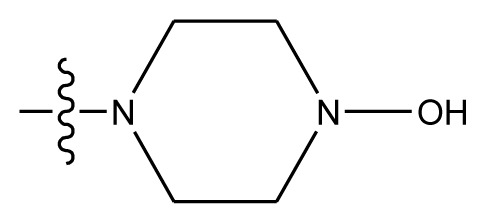	OCH_3_	OCH_3_	8.889	8.791
20-11	CN	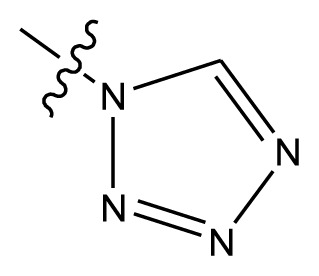	OCH_3_	OCH_3_	8.320	8.341
20-12	CN	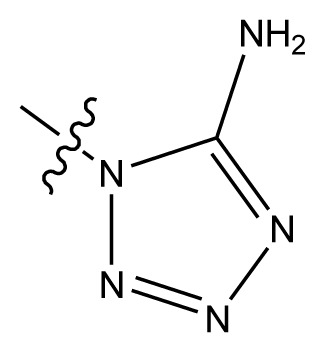	OCH_3_	OCH_3_	8.903	8.911
20-13	CN	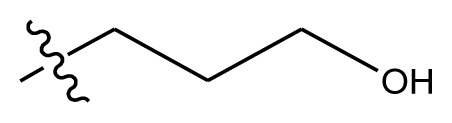	methylenedioxy		8.303	8.295
20-14	NO_2_	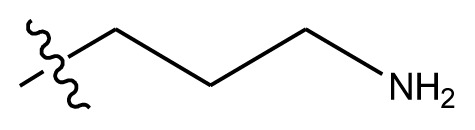	methylenedioxy		8.420	8.342
20-15	CN	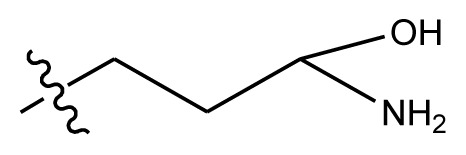	OCH_3_	OCH_3_	8.776	8.808

**Table 4:** The molecules of indenoisoquinoline derivatives.

Compd.	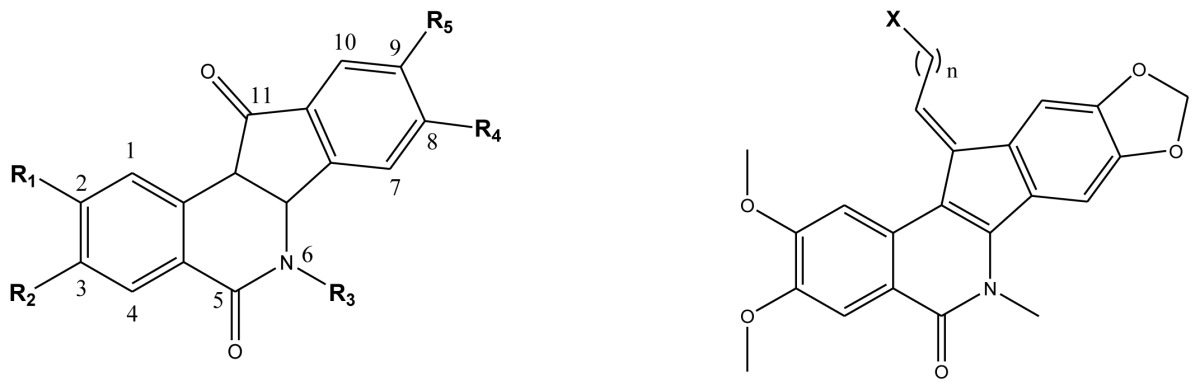				
	
	1–42			43–48	
	R_1_	R_2_	R_3_	R_4_	R_5_
1 *	OCH_3_	OCH_3_	CH_3_	methylenedioxy	
2	OCH_3_	OCH_3_	(CH_2_)_3_NH_2_	methylenedioxy	
3	OCH_3_	OCH_3_	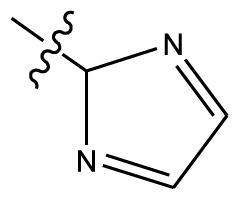	Methylenedioxy	
4	OCH_3_	OCH_3_	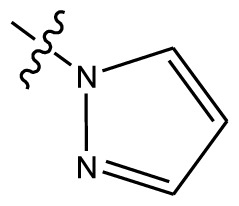	methylenedioxy	
5	OCH_3_	OCH_3_	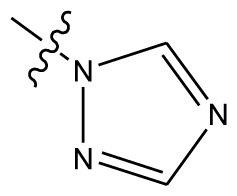	methylenedioxy	
6	OCH_3_	OCH_3_	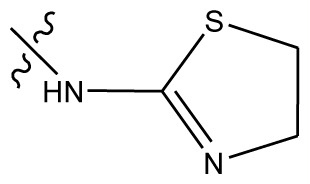	methylenedioxy	
7	OCH_3_	OCH_3_	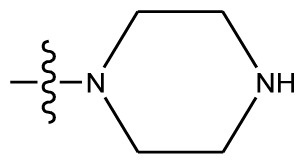	methylenedioxy	
8	OCH_3_	OCH_3_	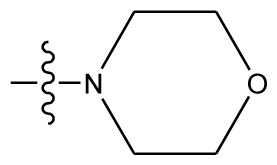	methylenedioxy	
9	OCH_3_	NO_2_	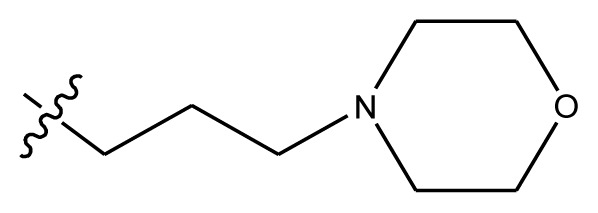	methylenedioxy	
10	OCH_3_	OCH_3_	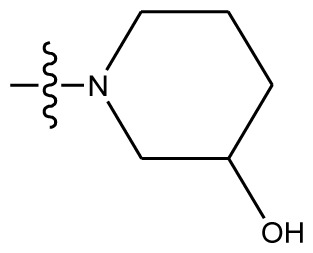	methylenedioxy	
11 *	OCH_3_	OCH_3_	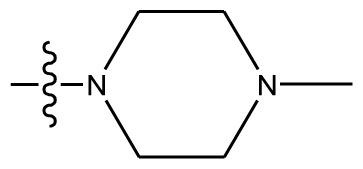	methylenedioxy	
12	OCH_3_	OCH_3_	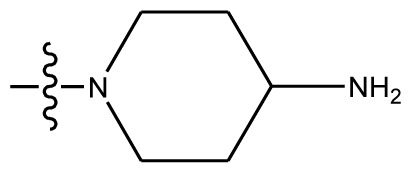	methylenedioxy	
13	OCH_3_	OCH_3_	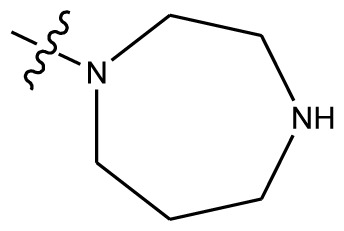	methylenedioxy	
14 *	OCH_3_	OCH_3_	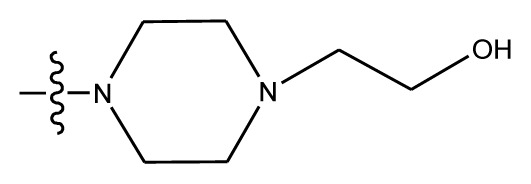	methylenedioxy	
15	OCH_3_	OCH_3_	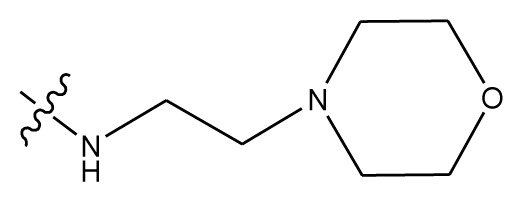	methylenedioxy	
16	H	H	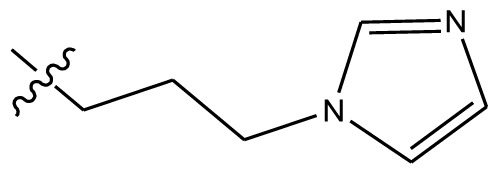	H	H
17 *	OCH_3_	OCH_3_	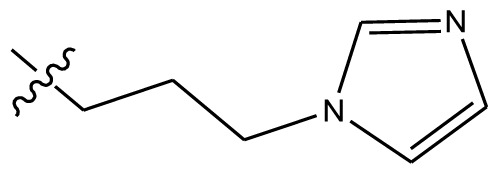	H	H
18	H	NO_2_	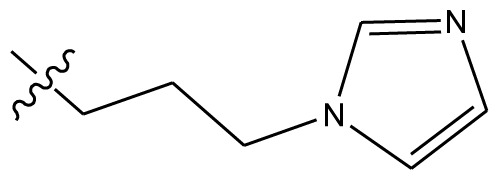	H	H
19	H	NO_2_	(CH_2_)_3_Cl	H	OCH_3_
20	H	NO_2_	(CH_2_)_3_NH_2_	H	OCH_3_
21	H	H	(CH_2_)_3_Br	H	H
22	H	H	(CH_2_)_3_NH_2_	H	H
23	H	H	(CH_2_)_3_N(CH_2_)_2_	H	H
24	H	NO_2_	(CH_2_)_3_N_3_	H	H
25	H	NO_2_	(CH_2_)_3_NH_2_	H	H
26	H	NO_2_	(CH_2_)_3_N(CH_2_)_2_	H	H
27 *	H	NO_2_	(CH_2_)_3_Br	H	H
28	H	H	(CH_2_)_3_N_3_	H	OCH_3_
29 *	H	H	(CH_2_)_3_NH_2_	H	OCH_3_
30	H	NO_2_	(CH_2_)_3_I	H	OCH_3_
31	H	H	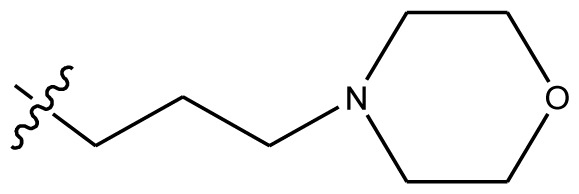	H	H
32	H	H	(CH_2_)_3_N_3_	H	H
33	H	NO_2_	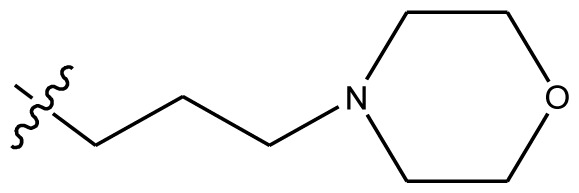	H	H
34	H	H	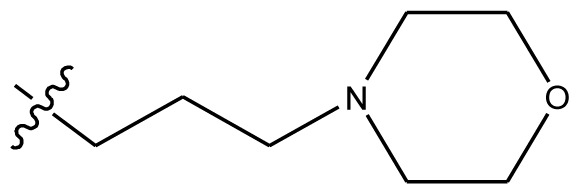	H	OCH_3_
35	H	NO_2_	(CH_2_)_3_NH(CH_2_)_3_OH	H	H
36	H	NO_2_	(CH_2_)_3_NH(CH_2_)_3_OH	H	OCH_3_
37	H	H	(CH_2_)_3_NH(CH_2_)_3_OH	H	OCH_3_
38	H	H	(CH_2_)_3_NH(CH_2_)_3_OH	H	H
39	H	NO_2_	(CH_2_)_3_N(CH_2_)_2_	H	OCH_3_
40	H	H	(CH_2_)_3_N(CH_2_)_2_	H	OCH_3_
41	H	NO_2_	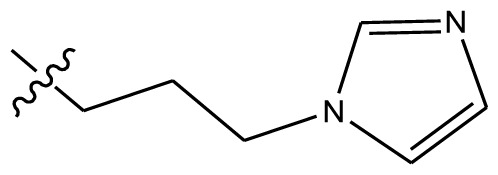	H	OCH_3_
42	H	H	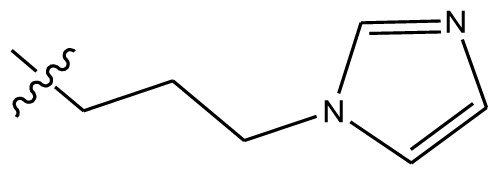	H	OCH_3_
**Compd.**	***n***	***X***	**Compd.**	***n***	***X***
43	3	Cl	46	5	Br
44	3	Br	47	3	I
45	4	Br	48 *	2	NH_2_

* Test set.

**Table 5:** Inhibitory activity and predicted values of indenoisoquinoline derivatives.

Comp. no.	Experiment (pGI50)	CoMFA		CoMSIA	

		Pred.	Res.	Pred.	Res.
1 *	4.168	4.003	0.165	4.335	−0.167
2	6.509	6.571	−0.062	6.679	−0.170
3	8.000	7.866	0.134	7.993	0.007
4	6.500	6.324	0.176	6.691	−0.191
5	7.071	7.206	−0.135	7.345	−0.274
6	8.000	8.113	−0.113	7.776	0.224
7	7.041	7.231	−0.190	7.135	−0.094
8	6.090	6.004	0.086	5.890	0.200
9	8.000	7.899	0.101	7.860	0.140
10	6.900	6.798	0.102	7.079	−0.179
11 *	4.939	5.067	−0.128	5.163	−0.224
12	6.590	6.497	0.093	6.889	−0.299
13	6.839	7.000	−0.161	7.132	−0.293
14 *	4.000	3.761	0.239	4.476	−0.476
15	5.830	5.812	0.018	6.337	−0.507
16	5.780	5.770	0.010	5.910	−0.130
17 *	8.000	8.198	−0.198	8.403	−0.403
18	7.824	7.793	0.031	7.850	−0.026
19	8.000	7.885	0.115	8.063	−0.063
20	8.000	8.145	−0.145	8.195	−0.195
21	5.155	4.996	0.159	4.666	0.489
22	6.796	6.689	0.107	6.535	0.261
23	6.041	6.003	0.038	5.990	0.051
24	4.140	4.095	0.045	4.003	0.137
25	6.991	7.023	−0.032	6.840	0.151
26	5.380	5.187	0.193	5.580	−0.200
27 *	4.000	3.695	0.305	4.443	−0.443
28	4.000	4.010	−0.010	4.147	−0.147
29 *	8.000	7.797	0.203	7.694	0.306
30	6.510	6.550	−0.040	6.443	0.067
31	4.670	4.535	0.135	4.871	−0.201
32	4.600	4.569	0.031	4.575	0.025
33	6.510	6.511	−0.001	6.505	0.005
34	4.070	4.034	0.036	4.104	−0.034
35	6.640	6.650	−0.010	6.497	0.143
36	7.921	7.905	0.016	8.133	−0.212
37	6.801	6.932	−0.131	7.004	−0.203
38	6.570	6.494	0.076	6.610	−0.040
39	8.000	8.049	−0.049	7.949	0.051
40	6.611	6.376	0.235	6.911	−0.300
41	8.000	8.001	−0.001	8.021	−0.021
42	6.170	6.003	0.167	6.250	−0.080
43	4.000	4.138	−0.138	4.517	−0.517
44	4.000	4.158	−0.158	4.231	−0.231
45	4.000	3.946	0.054	3.879	0.121
46	5.244	5.201	0.043	5.290	−0.046
47	5.056	4.875	0.181	4.756	0.300
48 *	6.237	6.040	0.197	6.660	−0.423

* Test set.
